# Mammalian Genes Preferentially Co-Retained in Radiation Hybrid Panels Tend to Avoid Coexpression

**DOI:** 10.1371/journal.pone.0032284

**Published:** 2012-02-24

**Authors:** Ben-Yang Liao, Andrew Ying-Fei Chang

**Affiliations:** Division of Biostatistics & Bioinformatics, Institute of Population Health Sciences, National Health Research Institutes, Zhunan, Miaoli County, Taiwan, Republic of China; University of Otago, New Zealand

## Abstract

Coexpression has been frequently used to explore modules of functionally related genes in eukaryotic genomes. However, we found that genetically interacting mammalian genes identified through radiation hybrid (RH) genotypes tend not to be coexpressed across tissues. This pattern remained unchanged after controlling for potential confounding factors, including chromosomal linkage, chromosomal distance, and gene duplication. Because >99.9% of the genetically interacting genes were identified according to the higher co-retention frequencies, our observation implies that coexpression is not necessarily an indication of the need for the co-presence of two genes in the genome, which is a prerequisite for cofunctionality of their coding proteins in the cell. Therefore, coexpression information must be applied cautiously to the exploration of the functional relatedness of genes in a genome.

## Introduction

Coexpression refers to the coherent transcription of genes in spatial, temporal, or environmental dimensions [1]–[3]. Presumably, proteins functioning together need to be co-present in a cell or tissue; production of an individual protein without its partners may lead to cell energy and material waste. Therefore, coexpression information has been frequently used to detect the functional modules of genes in the genome [4]–[6].

Proteins that are produced together undoubtedly require the co-presence of their coding genes in the genome. However, regulation of protein abundance does not necessarily occur at the transcriptional level [7], [8], and gene expression does not determine the fate of tissue differentiation [9]. In addition, analyses in mammals [10], nematodes [11], and flies [12] indicate that many coexpressed gene clusters are unlikely to have originated to optimize gene regulation. Consequently, it remains elusive whether the requirement for the co-presence of two genes in a genome is reflected by an increased level of coexpression and, therefore, whether coexpression predicts the cofunctionality of genes.

To understand the biological implications of gene coexpression, we examined whether elevated coexpression predicts the need for the co-presence of genes in the genome, which is a prerequisite for the cofunctionality of their protein products. Exploiting genotypes of human, mouse, rat, and dog radiation hybrid (RH) panels, researchers recently calculated the co-retention frequencies of all mammalian gene pairs with an intergenic distance (*D*, see Methods) of ≥10 megabases (Mb) in the human genome, which resulted in the identification of >7×10^6^ “genetic interactions” among >18,000 genes [13]. Because >99.9% of these interactions were identified through higher co-retention frequencies than by chance, such interactions can be considered as an index for the tendency of two genes to be co-present in the genome. In addition, because the topology of the resulted interaction network suggests the comprehensiveness of the interactions identified, the catalog of interactions is ideal for us to perform systematic analyses without inspection biases [14]–[16]. To our surprise, genes that were preferentially co-retained in the genome consistently showed lower coexpression compared to other gene pairs. This finding suggests that coexpression information must be used cautiously in the exploration of the functional relatedness of genes in a genome.

## Results and Discussion

We measured coexpression between two genes from expression profile similarities across 63 human or 58 mouse tissues, using the equation *ln*[(1+*CoExp*)/(1−*CoExp*)] (see Materials and Methods). Larger values of *ln*[(1+*CoExp*)/(1−*CoExp*)] indicate higher coexpression. If coexpression predicts preference for the co-presence for two genes in the mammalian genome, then higher coexpression (and, hence, larger *ln*[(1+*CoExp*)/(1−*CoExp*)]) is expected to be found in pairs of “genetically interacting genes,” as defined in Lin et al. (2010) (hereafter referred to as “co-retained gene pairs” [*CRGPs*]), than in other “non-co-retained” gene pairs (*nCRGPs*) (Fig. S1).

**Figure 1:**
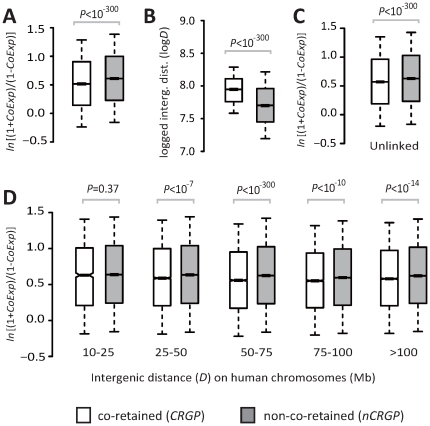
Coexpression in and characteristics of chromosomal linkage of *CRGPs* vs. *nCRGPs*. Box plots of *ln*[(1+*CoExp*)/(1−*CoExp*)] of *CRGPs* vs. *nCRGPs* in (**A**) all gene pairs, (**C**) unlinked gene pairs, and (**D**) linked gene pairs with specified ranges of *D*. *CoExp* is measured by Spearman's ρ of expression levels between genes across human tissues (see Fig. S2 for *CoExp* measured by Pearson's *r*). (**B**) Box plots of log*D* of linked *CRGP* vs. linked *nCRGPs*. Upper quartile, median, and lower quartile values are indicated in each box. Bars outside the box indicate semi-quartile ranges. *P*-values are from a Mann-Whitney *U* test.

Our initial analysis revealed that *ln*[(1+*CoExp*)/(1−*CoExp*)] values between *CRGPs* were significantly lower than those between *nCRGPs* (*P*<10^−300^, Mann-Whitney *U* test; Fig. 1A). However, chromosomal linkage promotes coexpression: *D* between linked genes is negatively correlated with their coexpression, even when *D* is on the order of tens of Mb in length [10]. Our result showed that the proportion of gene pairs located on the same human chromosome (linked) for *CRGPs* (61,986/2,615,153 = 2.43%) was only approximately half of that for *nCRGPs* (2,234,672/49,999,275 = 4.47%) (*P*<10^−300^, χ^2^ test). Compared to linked *nCRGPs*, the linked *CRGPs* had significantly larger *D* values (*P*<10^−300^, *U* test; Fig. 1B) in the human genome.

We can potentially explain the tendencies of *CRGPs* to be unlinked, or to have a larger *D* when linked, in two ways. First, *CRGPs* are more subject to transcriptional interference [10]. The human genome has evolutionarily shaped its architecture to avoid the deleterious effects of transcriptional interference [10]. Second, the “genetic interaction” data obtained by Lin *et al.* (2010) poses intrinsic biases in chromosomal linkage. Regardless of the cause, the bias in chromosomal linkage is an important factor that needs to be controlled in our analyses.

To determine whether a lower frequency of chromosomal linkage or larger *D* of linked genes sufficiently explains the lower coexpression of *CRGPs* (Fig. 1A), we classified all gene pairs into linked and unlinked groups (those located on different chromosomes), on the basis of their coordinates on the human genome. We further categorized linked gene pairs into 5 groups with similar *D* values, to control for *D* (Fig. 1D). For unlinked genes, the *CRGPs* still showed significantly lower *ln*[(1+*CoExp*)/(1−*CoExp*)] values than did the *nCRGPs* (*P*<10^−300^, *U* test; Fig. 1C); for linked gene pairs, *CRGPs* also consistently showed significantly lower *ln*[(1+*CoExp*)/(1−*CoExp*)] values than *nCRGPs* in nearly all groups (*P*≤10^−7^, *U* test; Fig. 1D), except for the group of 10–25 Mb (*P* = 0.37, *U* test; Fig. 1D). Hence, lower coexpression of *CRGPs* cannot be explained by chromosomal linkage or *D*. Duplicate genes share similarity in expression by ancestry and, thus, may confound our result [2], [17]. However, the removal of paralogous gene pairs from the analysis produced a virtually identical result (Fig. S3, S4), suggesting that lower coexpression between *CRGPs* is unrelated to gene duplication.

To determine whether our observations are specific to human, a parallel analysis was conducted on mouse data (see Materials and Methods). We used the mouse genes that are one-to-one orthologs to human genes mapped in Lin *et al.* (2010). When linkage was defined by mouse genome coordinates and coexpression was measured based on expression levels across 58 mouse tissues, the result remained consistent with Fig. 1, although the statistical significance of some of the comparisons was reduced (Fig. S5).

**Figure 2:**
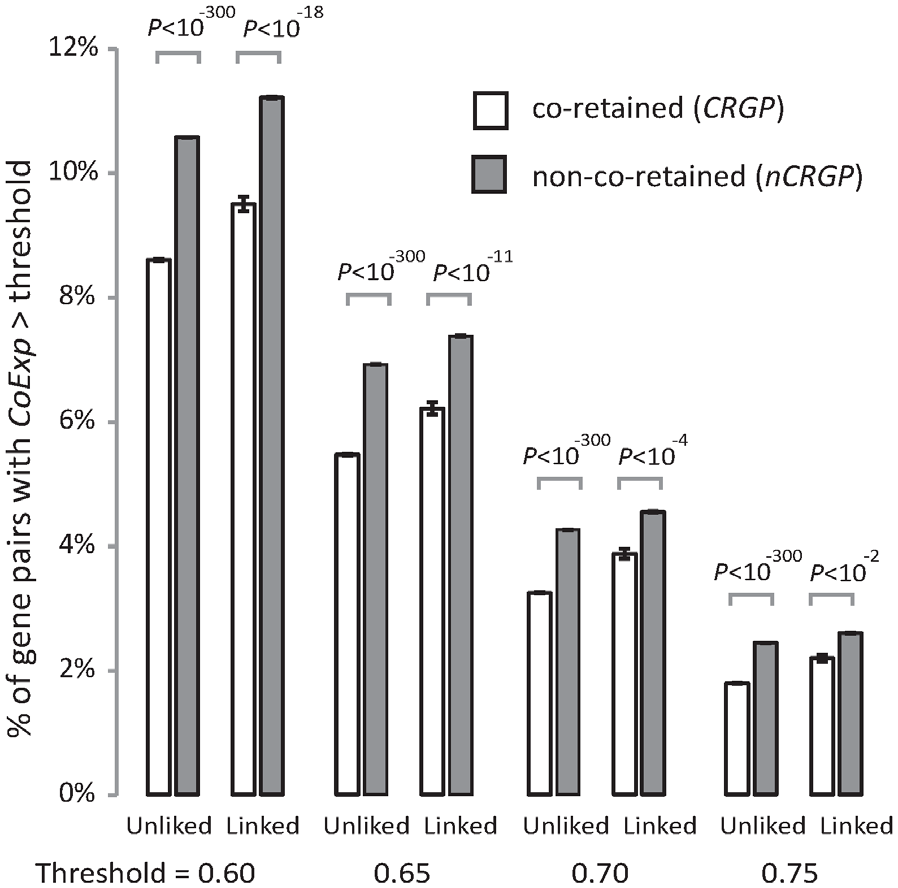
*nCRGPs* comprise a higher percentage of highly coexpressed genes. Compared with *nCRGPs*, *CRGPs* have a lower percentage of gene pairs with high coexpression, as defined by the threshold of *CoExp* shown in the bottom, after controlling for chromosomal linkage. *CoExp* is measured by Spearman's ρ of expression levels between genes (see Fig. S6 when *CoExp* was measured by Pearson's *r*). Error bars show one standard error of the proportion. *P*-values are from a χ^2^ test.

In several previous studies [4]–[6], [18], researchers have claimed that coexpression must be sufficiently high to be considered “biologically relevant” and to be used in exploring the functional relatedness of genes [19]. Hence, we examined coexpression from the aspect of frequencies of genes with high coexpression in the group. We used different thresholds of *CoExp* to define high coexpression (*CoExp*≥0.6, 0.65, 0.7, or 0.75) [3]. Linked and unlinked genes were separated to control for bias in the chromosomal linkage. As shown in Fig. 2, regardless of the threshold used, *CRGPs* consistently showed a lower proportion of highly coexpressed gene pairs than *nCRGPs*. Parallel analysis with mouse genome coordinates and gene expression data generated a result consistent with Fig. 2 (Fig. S7).

In summary, our analysis showed no indication that coexpression between genes indicates a need for co-presence in the genome. In contrast, we consistently observed patterns indicating that co-retained genes tend to avoid coexpression in both human and mouse genomes. The result presented in this study implies that factors unrelated to functionality (*e.g.* transcriptional interference [10]), may cause the coexpression of mammalian genes. Because high coexpression between most of the coexpressed gene pairs is not necessarily evolutionarily conserved, our study implies that it is perhaps evolutionary conservation of coexpression [5], and not coexpression itself, that predicts cofunctionality.

## Materials and Methods

*CRGPs*, which were defined as human genes with “genetic interactions” based on their having RH genotypes with an FDR threshold of ≤0.05, were obtained from the supplementary materials of [13]. The use of a more stringent FDR threshold (≤0.001) to define *CRGPs* and *nCRGPs* did not change the results of the analysis (Fig. S8). Chromosomal coordinates, one-to-one orthologs, and annotations of paralogous relationships of human and mouse genes based on Ensembl v62 were retrieved through BioMart (http://www.biomart.org/). The intergenic distance *D* was calculated as the distance in nucleotides between the transcriptional start sites of two genes.

Expression levels in 63 normal human tissues or 58 normal mouse tissues were obtained from Gene Atlas v2 [20] following a previous study [10]. Only 10,313 human genes with genetic interaction data, Ensembl annotations, and microarray data were used (Fig. S1). *CoExp* was defined by the Spearman's correlation coefficient (ρ) or Pearson's correlation coefficient (*r*) of expression levels across human tissues. Because *CoExp* measured by Spearman's ρ yielded statistically more significant results, those results are presented in the main text. Consistent results derived from using Pearson's *r* to calculate *CoExp* are shown as Figs. S2 and S6.

In addition to *CoExp*, the expression profile “dissimilarity” between genes was calculated from the Euclidean distance 
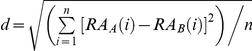
 [21], where *n* is the number of tissues, and 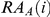 or 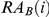
is the relative transcriptional abundance of gene *A* or gene *B*, respectively, in tissue *i*. The relative transcriptional abundance was calculated from the expression level of a gene in the tissue examined divided by the summation of expression levels of that gene in all of the tissues in the dataset [21]. A lower *d* indicates a higher level of coexpression. Use of *d* yielded a result (Fig. S9) that was consistent with the result based on *CoExp* (Fig. 1), suggesting the robustness of the conclusion reached.

## Supporting Information

Figure S1 **Flow chart** illustrating the processes used to generate *CRGPs* and *nCRGPs* for comparisons in co-expression.
(PDF)

Figure S2 **Regenerated** **Figure 1** when *CoExp* is calculated by Pearson's *r* of expression levels between genes. See legend of Fig. 1 for detailed description.
(PDF)

Figure S3 **Regenerated (A)** **Fig. 1C and (B)** **Fig. 1D** by excluding gene pairs that are paralogous from the analysis.
(PDF)

Figure S4 **Regenerated (A) Fig. S2C and (B)Fig. S2D** by excluding gene pairs that are paralogous from the analysis.
(PDF)

Figure S5 **Regenerated** **Fig. 1** when *CoExp* is measured using mouse gene expression data and linkage and *D* are defined using mouse genome coordinates. See legend of Fig. 1 for detailed description.
(PDF)

Figure S6 **Regenerated** **Fig. 2** when *CoExp* is calculated by Pearson's *r* of expression levels between genes. See legend of Fig. 2 for detailed description.
(PDF)

Figure S7 **Regenerated** **Fig. 2** when *CoExp* is measured using mouse microarray data and linkage and *D* are defined using mouse genome coordinates. See legend of Fig. 2 for detailed description.
(PDF)

Figure S8 Regenerated Fig. 1 using a more stringent FDR threshold (≤0.001) to define *CRGPs* and *nCRGPs*. See legend of Fig. 1 for detailed description.
(PDF)

Figure S9 Regenerated Figure 1 when expression dissimilarity is calculated by *d*, the Euclidean distance of the relative transcriptional abundance between genes. A lower *d* indicates a higher level of coexpression. See legend of Fig. 1 for detailed description.
(PDF)
