# Supplementary material for: Mammalian Genes Preferentially Co-Retained in Radiation Hybrid Panels Tend to Avoid Coexpression
Source: PLoS One. 2012 Feb 24;7(2):e32284. doi: 10.1371/journal.pone.0032284 (PMC3286474; doi:10.1371/journal.pone.0032284)
Supplement: Figure S1 — Flow chart illustrating the processes used to generate CRGPs and nCRGPs for comparisons in co-expression. (PDF) [file pone.0032284.s001.pdf]

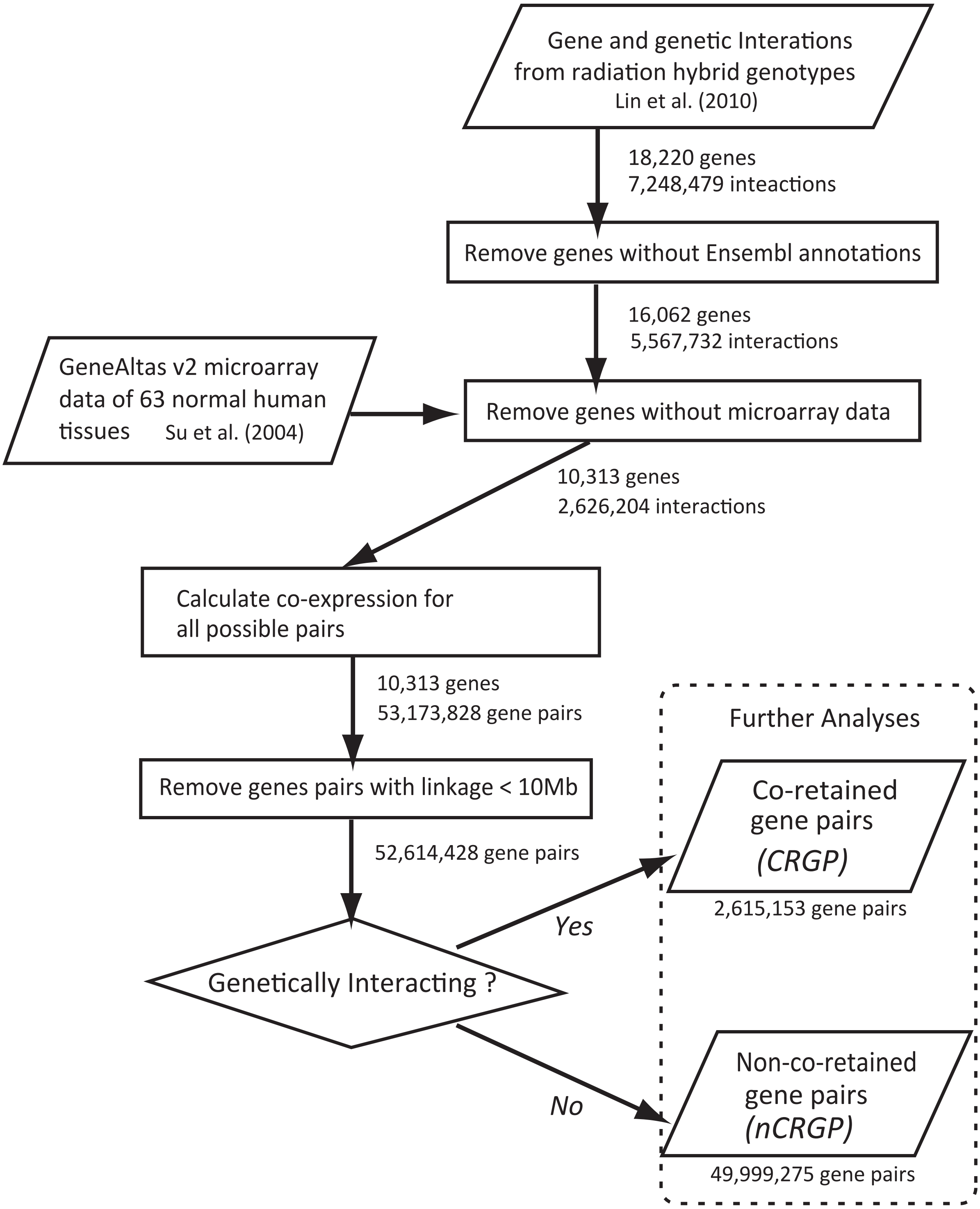

Figure S1. Flow chart illustrating the processes used to generate *CRGPs* and *nCRGPs* for comparing coexpression.
