# Supplementary material for: Mammalian Genes Preferentially Co-Retained in Radiation Hybrid Panels Tend to Avoid Coexpression
Source: PLoS One. 2012 Feb 24;7(2):e32284. doi: 10.1371/journal.pone.0032284 (PMC3286474; doi:10.1371/journal.pone.0032284)
Supplement: Figure S2 — Regenerated Figure 1 when CoExp is calculated by Pearson's r of expression levels between genes. See legend of Fig. 1 for detailed description. (PDF) [file pone.0032284.s002.pdf]

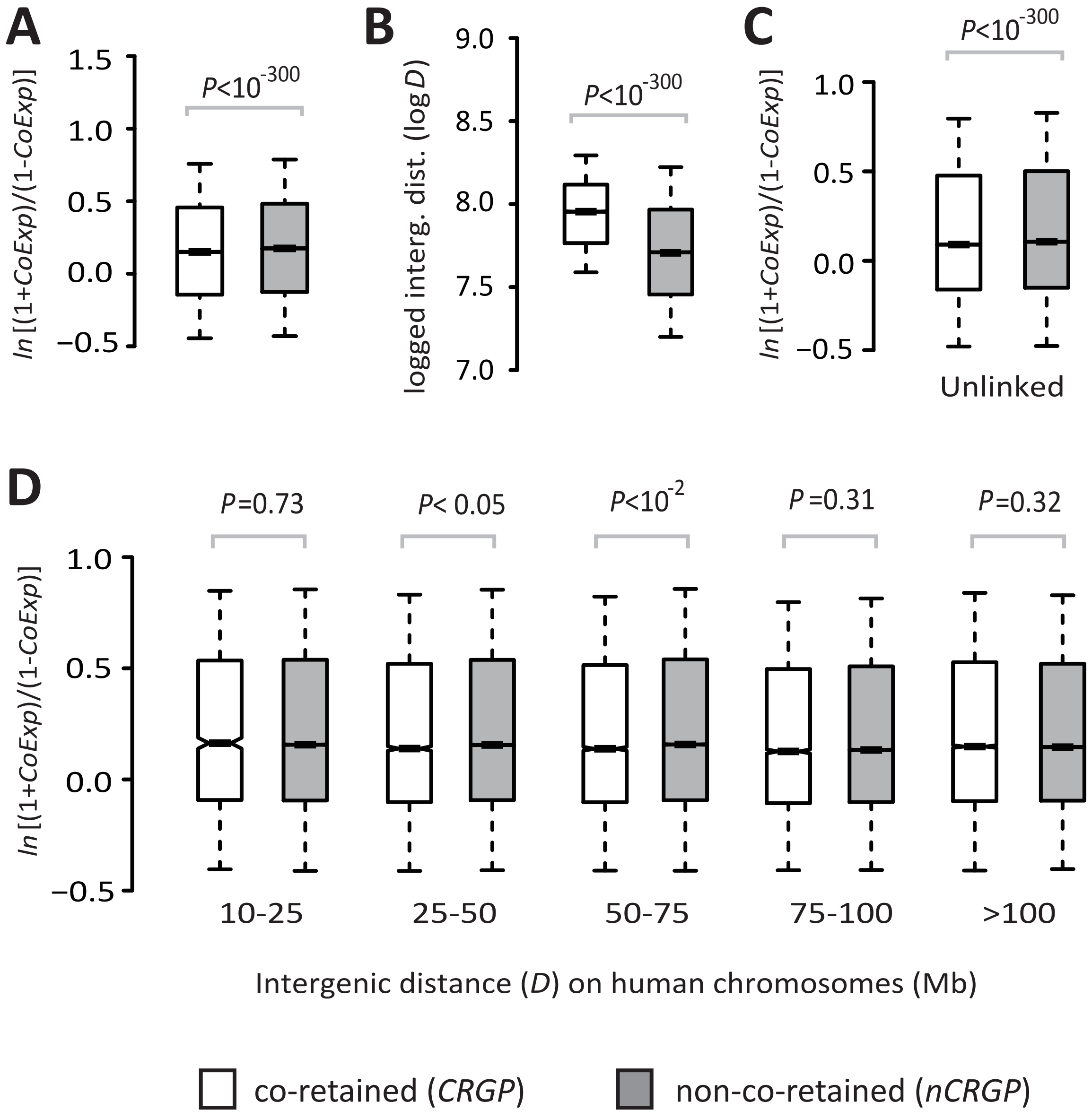

Figure S2. Regenerated Fig. 1 obtained by calculating *CoExp* with Pearson's *r* of expression levels between genes. See legend of Fig. 1 for detailed description.
