# Supplementary material for: Mammalian Genes Preferentially Co-Retained in Radiation Hybrid Panels Tend to Avoid Coexpression
Source: PLoS One. 2012 Feb 24;7(2):e32284. doi: 10.1371/journal.pone.0032284 (PMC3286474; doi:10.1371/journal.pone.0032284)
Supplement: Figure S3 — Regenerated (A) Fig. 1C and (B) Fig. 1D by excluding gene pairs that are paralogous from the analysis. (PDF) [file pone.0032284.s003.pdf]

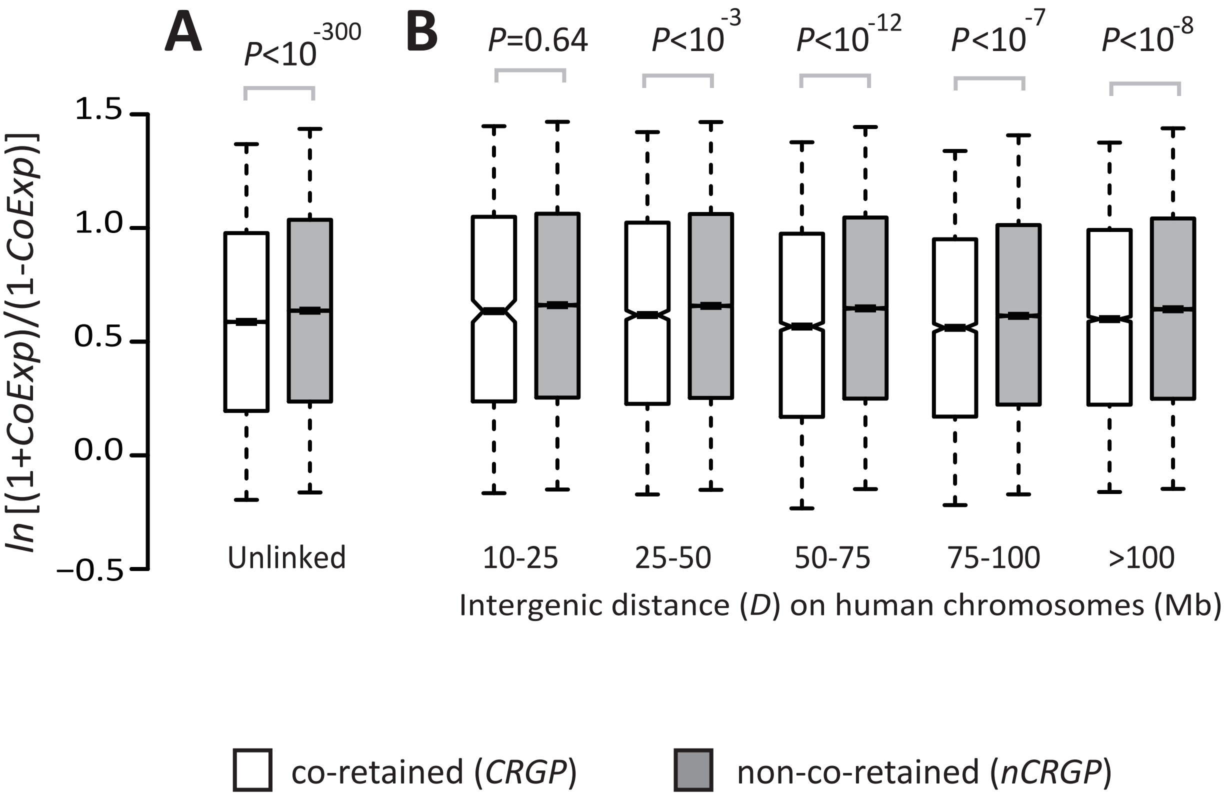

Figure S3. Regenerated (A) Fig. 1C and (B) Fig. 1D obtained by excluding paralogous gene pairs from the analysis.
