# Supplementary material for: Mammalian Genes Preferentially Co-Retained in Radiation Hybrid Panels Tend to Avoid Coexpression
Source: PLoS One. 2012 Feb 24;7(2):e32284. doi: 10.1371/journal.pone.0032284 (PMC3286474; doi:10.1371/journal.pone.0032284)
Supplement: Figure S4 — Regenerated (A) Fig. S2C and (B)Fig. S2D by excluding gene pairs that are paralogous from the analysis. (PDF) [file pone.0032284.s004.pdf]

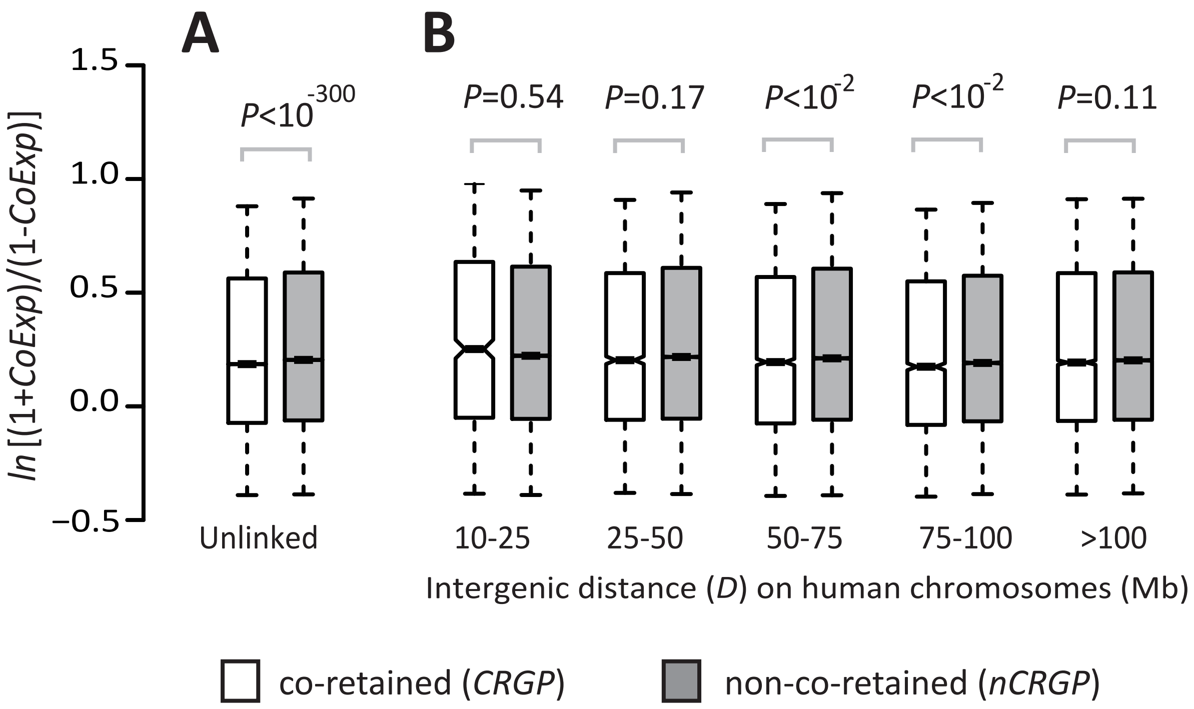

Figure S4. Regenerated (A) Fig. S2C and (B) Fig. S2D obtained by excluding paralogous gene pairs from the analysis.
