# Supplementary material for: Mammalian Genes Preferentially Co-Retained in Radiation Hybrid Panels Tend to Avoid Coexpression
Source: PLoS One. 2012 Feb 24;7(2):e32284. doi: 10.1371/journal.pone.0032284 (PMC3286474; doi:10.1371/journal.pone.0032284)
Supplement: Figure S5 — Regenerated Fig. 1 when CoExp is measured using mouse gene expression data and linkage and D are defined using mouse genome coordinates. See legend of Fig. 1 for detailed description. (PDF) [file pone.0032284.s005.pdf]

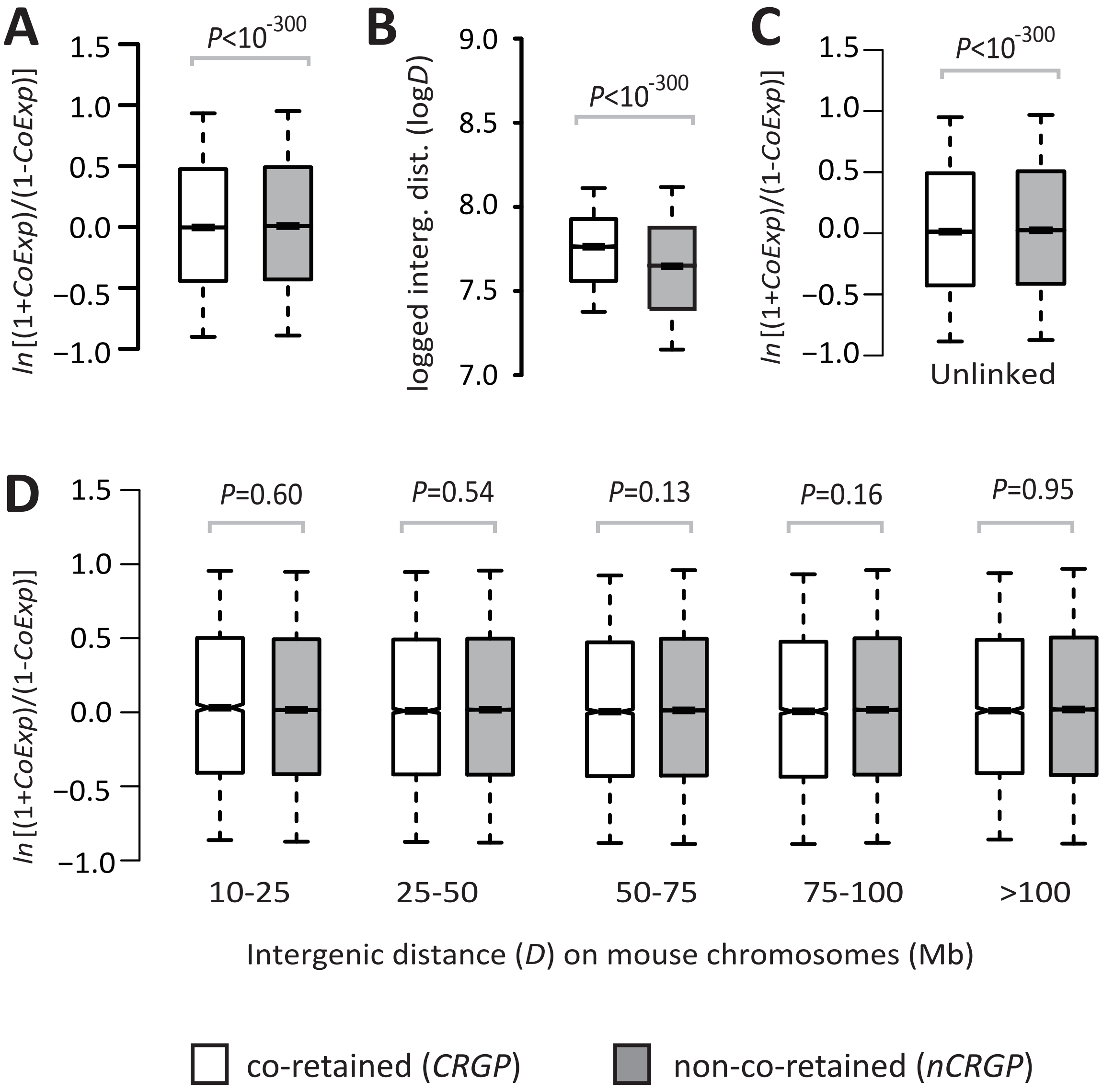

Figure S5. Regenerated Fig. 1 obtained by measuring *CoExp* from mouse gene expression data and by defining linkage and *D* from mouse genome coordinates. See legend of Fig. 1 for detailed description.
