# Supplementary material for: Mammalian Genes Preferentially Co-Retained in Radiation Hybrid Panels Tend to Avoid Coexpression
Source: PLoS One. 2012 Feb 24;7(2):e32284. doi: 10.1371/journal.pone.0032284 (PMC3286474; doi:10.1371/journal.pone.0032284)
Supplement: Figure S7 — Regenerated Fig. 2 when CoExp is measured using mouse microarray data and linkage and D are defined using mouse genome coordinates. See legend of Fig. 2 for detailed description. (PDF) [file pone.0032284.s007.pdf]

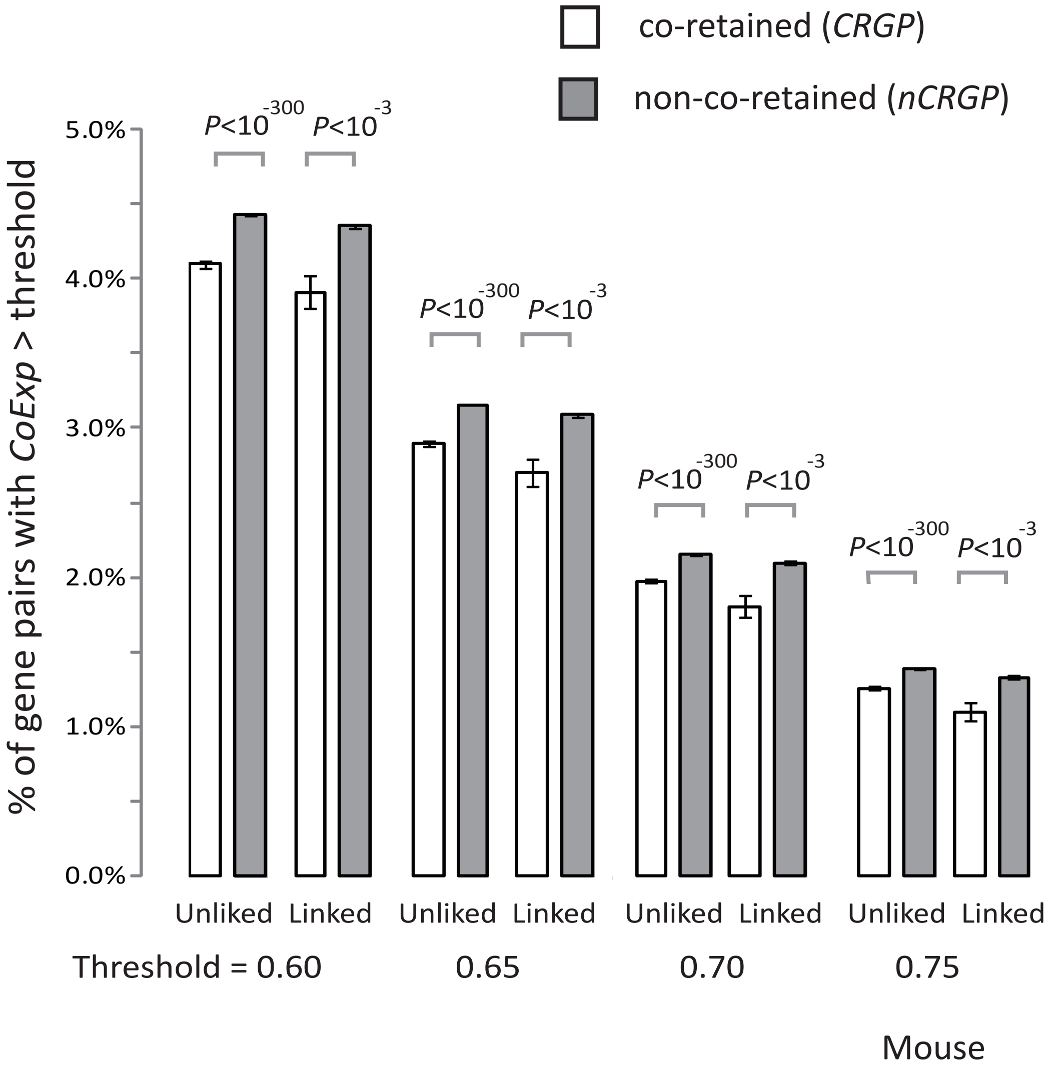

Figure S7. Regenerated Fig. 2 obtained by measuring *CoExp* from mouse microarray data and by defining linkage and *D* with mouse genome coordinates. See legend of Fig. 3 for detailed description.
