# Supplementary material for: Mammalian Genes Preferentially Co-Retained in Radiation Hybrid Panels Tend to Avoid Coexpression
Source: PLoS One. 2012 Feb 24;7(2):e32284. doi: 10.1371/journal.pone.0032284 (PMC3286474; doi:10.1371/journal.pone.0032284)
Supplement: Figure S8 — Regenerated Fig. 1 using a more stringent FDR threshold (≤0.001) to define CRGPs and nCRGPs. See legend of Fig. 1 for detailed description. (PDF) [file pone.0032284.s008.pdf]

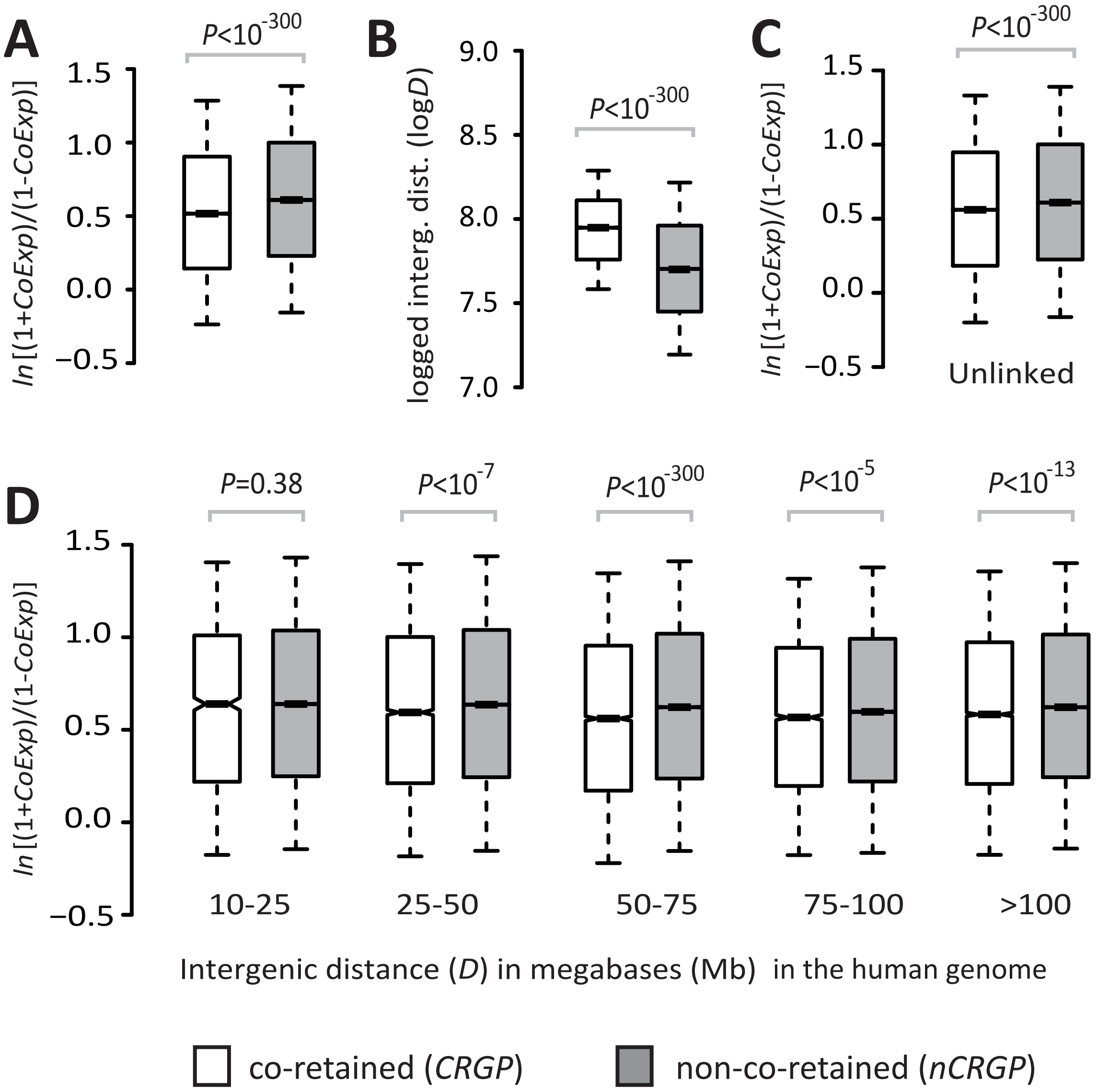

Figure S8. Regenerated Fig. 1 obtained by using a stringent FDR threshold (≤0.001) to define *CRGPs* and *nCRGPs*. See legend of Fig. 1 for detailed description.
