# Supplementary material for: Mammalian Genes Preferentially Co-Retained in Radiation Hybrid Panels Tend to Avoid Coexpression
Source: PLoS One. 2012 Feb 24;7(2):e32284. doi: 10.1371/journal.pone.0032284 (PMC3286474; doi:10.1371/journal.pone.0032284)
Supplement: Figure S9 — Regenerated Figure 1 when expression dissimilarity is calculated by d, the Euclidean distance of the relative transcriptional abundance between genes. A lower d indicates a higher level of coexpression. See legend of Fig. 1 for detailed description. (PDF) [file pone.0032284.s009.pdf]

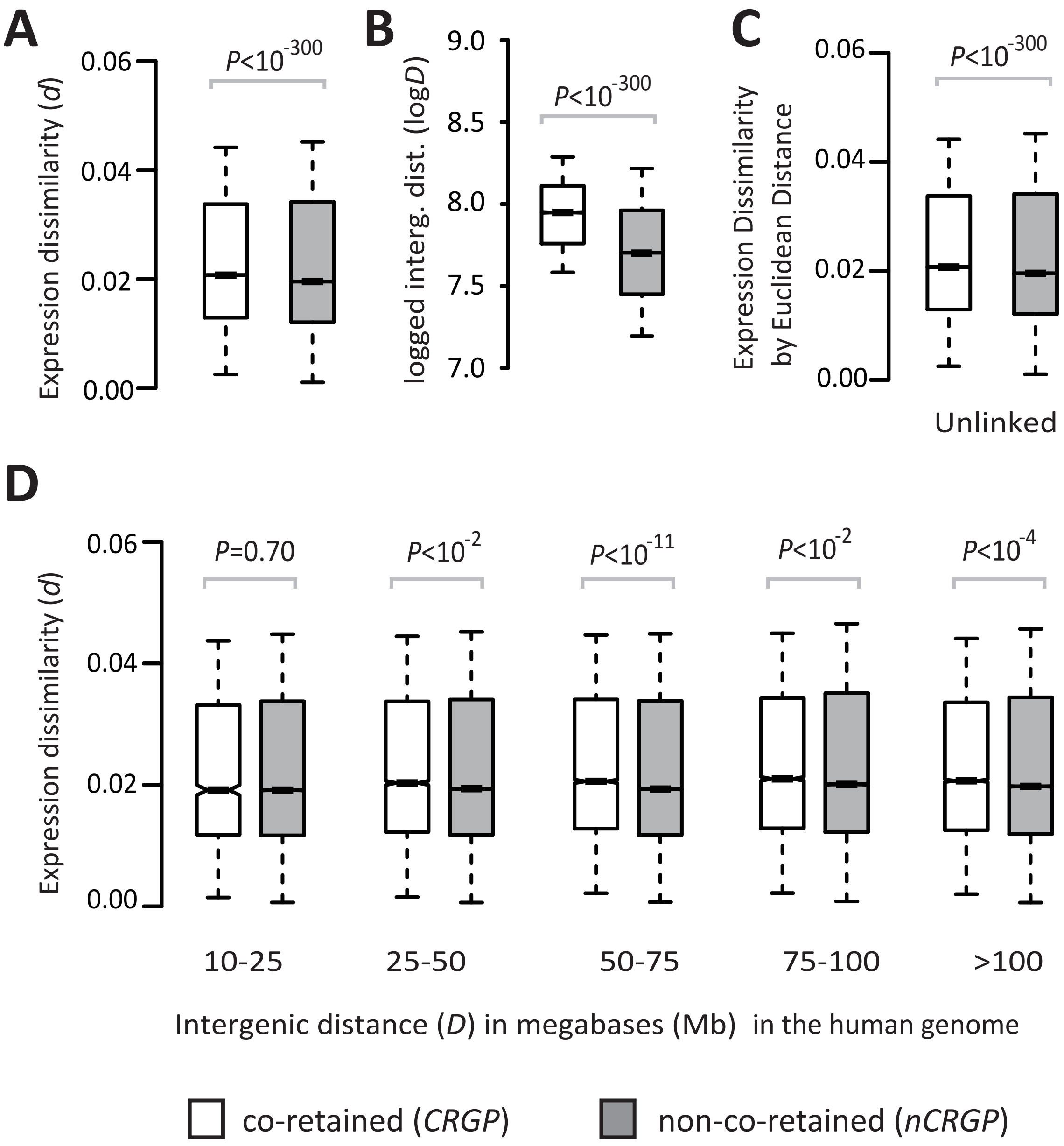

Figure S9. Regenerated Fig. 1 obtained by calculating expression dissimilarity with *d*, which is the Euclidean distance of the relative transcriptional abundance between genes. Lower *d* indicates higher level of coexpression. See legend of Fig. 1 for detailed description.
